# Epstein-Barr Virus and Cytomegalovirus induced Acute Hepatitis in Young Female Patient

**DOI:** 10.5005/jp-journals-10018-1134

**Published:** 2015-01-06

**Authors:** İhsan Ates, Mustafa Kaplan, Nisbet Yilmaz, Filiz Çiftçi

**Affiliations:** 1 Department of Internal Medicine, Ankara Numune Education and Research Hospital, Sihhiye, Ankara, Turkey; 2 Department of Infectious Diseases and Clinical Microbiology, Ankara Numune Education and Research Hospital, Sihhiye, Ankara, Turkey

**Keywords:** Acute hepatitis, CMV, EBV.

## Abstract

Acute hepatitis is a disorder that goes with liver cell necrosis and liver inflammation. Among the causes of acute hepatitis, the most common reasons are viral hepatitis. About 95% of the acute hepatitis generate because of hepatotropic viruses. Epstein-barr virus (EBV) and cytomegalovirus (CMV) are from the family of herpes viruses and rare causes of acute hepatitis. In this case report, acute hepatitis due to EBV and CMV coinfection will be described.

**How to cite this article:**

Ates İ, Kaplan M, Yilmaz N, Çiftçi F. Epstein-Barr Virus and Cytomegalovirus induced Acute Hepatitis in Young Female Patient. Euroasian J Hepato-Gastroenterol 2015;5(1):60-61.

## INTRODUCTION

In Asia and some European countries, the most common cause of acute hepatitis is hepatitis viruses.^1^ It has been seen that many viruses cause acute hepatitis. Although the most common causative viruses are hepatitis A, B, C, D and E, it was shown that herpes simplex virus, varicella-zoster virus, adenovirus, epstein-barr virus (EBV) and cytomegalovirus (CMV) can rarely cause acute hepati-tis.^2^ Although mild elevations in liver enzymes can be seen in 40 to 80% of cases due to EBV-induced infectious mononucleosis (EM), clinical jaundice occures in 5 to 11% of cases.^3^ Liver test abnormality is seen in only symptomatic CMV infection. Transaminases may increase up to five times in these cases.^4,5^ Unlike other herpes viruses, jaundice does not develop in CMV-induced hepatitis. In this case report, a patient with acute hepatitis due to CMV and EBV coinfection will be presented.

## CASE REPORT

A 28-year-old female patient was admitted to the emergency department with fever, fatigue, nausea. Although she used amoxicillin-clavulanic acid (1 gm) for 3 days due to high fever associated with malaise, her fever continued. She was hospitalized with a preliminary diagnosis of toxic hepatitis. A chronic systemic disease, smoking, alcohol, drug use could not be found in this patient.

On physical examination, fever was 37.8°C, pulse was 105/min, arterial blood pressure was 100/60 mm Hg, respiratory rate was 18/min, oropharynx was hyper-emic, there was 3 to 4 painful lymphadenopathies (LAP) (4-6 mm in diameter) at the beginning of scalp behind the right ear. There was minimal hepatosplenomegaly (HSM). Other findings were normal. In laboratory tests WBC: 10.78 × 10^3^/μl, neutrophil could not be detected properly. The levels of other parameters are given; lymphocytes; 6.22 × 10^3^/μl (1.3 to 3.5), hemoglobulin; 14.2 gm/dl, platelet: 174 × 10^3^/μl, alanine aminotransferase (ALAT); 742 IU/l (10-50), aspartate aminotransferase (AST); 517 IU/l (10-50), alkaline phosphatase (ALP); 394 IU/l (40-130), GGT: 404 IU/l (10-71), total bilirubin; 6.7 mg/dl (<1.4), direct bilirubin; 4.73 mg/dl (<0.3), erythrocyte sedimentation rate (ESR); 19 mm (0-20), C-reactive protein (CRP): 14.54 mg/dl (< 5). There was a predominance of atypical lymphocytes in the peripheral blood smear. Upper abdomen ultrasonography which was done to investigate the etiology of LFT disorder was normal except minimal HSM. Antimitochondrial antibody, antinuclear antibody, antismooth muscle antibodies, antineutrophil cytoplasmic antibody, acute and chronic viral hepatitis markers were normal. Serological tests for salmonella and brucella were negative. In addition, iron, iron binding capacity, ferritin, 24-hour urine copper, ceruloplasmin, alpha-fetoprotein, alpha-1 antitrypsin levels were within normal limits. EBV parameters like EBV VCA IgM, VCA IgG and EBNA IgM were positive, also, CMV IgM was positive with high-titer. Cytomegalo-virus IgG titer and other parameters were negative. According to these findings, patient was diagnosed as acute hepatitis due to the EBV and CMV coinfection. Supportive treatment was given, liver function tests (LFTs) were normalized gradually after 3 weeks.

## DISCUSSION

Epstein-barr virus is a member of the herpes virus family, more than 90% of the adult population has encountered this virus.^6^ Transmission of infections is with close contact in children, with kissing in adults. Also, blood-related transmission has been documented.^7^ Cytomegalovirus infection causes EM disease which is asymptomatic and subclinically in childhood and symptomatic (like fever, sore throat, lymphadenopathy) in adulthood.^8^ Although liver involvement is common, hepatomegaly is seen only in 10 to 15% of cases, spontaneously recovering elevated liver enzymes is seen in 80 to 90% of patients. LFT elevations are generally less than 5 times, bilirubin levels rise less than 40 % of the basal value. Other etiological factors must be investigated when ten times higher values are seen.^9^ Our case was 28 years old and admitted with fever, sore throat and LAP. There was more than ten-fold elevation in some liver enzymes. In etiologic investigations, EBV VCA IgM, EBNA IgM, CMV IgM and CMV DNA were positive. With these findings acute hepatitis due to co-infection with CMV and EBV were considered in our patient. Cytomegalovirus is another member of the herpes virus family and may be asymptomatic or cause EM in children and adults. It is known that EM is originated 79% from EBV and 21% from CMV. It is difficult to distinguish between EBV and CMV-related EM, but CMV-related infections are mostly seen post-transfusional and features, such as pharyngitis and lymphadenopathy are detected with a lesser extent. For the strict separation of the two factors, serological tests are more frequently needed.^10^ In CMV related EM, although a self-limiting LFT disorder is seen in 90% of patients, an extreme elevation is not seen.^5,11^ Alanine aminotrans-terase elevation is more common than AST elevation but elevation of total bilirubin and ALP are rare.^12^ Because of coinfection at the same time, transaminases rose more than 15 times and ALP and bilirubin levels rised six times. Alanine aminotransferase elevation was more dominant than AST elevation. In the presence of both infection, granulomatous hepatitis and acute liver failure are very rare. In our case, LFTs and bilirubin levels decreased to normal limits after 4 weeks symptomatic and supportive treatment.

## CONCLUSION

A rare cause of acute hepatitis is EBV and CMV coinfection and should be kept in mind in patients who presented with abnormal liver function tests. Epstein-barr virus and CMV coinfection should not be ignored in cases when considering etiologic factors of elevation in LFTs more than ten times.
